# Elements of perceived good physician leadership and their relation to leadership theory

**DOI:** 10.1108/LHS-01-2021-0002

**Published:** 2021-08-31

**Authors:** Sari Huikko-Tarvainen

**Affiliations:** School of Business and Economics, University of Jyväskylä, Jyväskylä, Finland

**Keywords:** Physician leadership, Leadership theory

## Abstract

**Purpose:**

This research paper aims to discover the elements of good physician leadership as perceived by physicians and to find out how the findings connect to the leadership theory.

**Design/methodology/approach:**

The subjects (*n* = 50) of this qualitative study are physicians from four hierarchical levels (residents/specialising physicians, specialists, heads of departments and chief physicians). Content analysis with a constructivist-interpretative approach by thematisation was the chosen method, and it was also analysed how major leadership theories relate to good physician leadership.

**Findings:**

Physician leaders are expected to possess the professional skills of physicians, understand how the work affects physicians’ lives and be competent in applying suitable leadership approaches following different situations and people. Trust, fairness, empathy, social skills, two-way communication skills, regular feedback, collegial respect and emotional intelligence are expected. As medical expertise connects leaders and followers, success in medical leadership comes from credibility in medical expertise, making medical leadership an inseparable part of good physician leadership. Subordinates are physician colleagues, who have their informal leadership roles on their hierarchical levels, making physician leadership a multidimensional leadership setting wherein formal leaders lead informal leaders, which blurs the traditional leader–follower boundary. In summary, good physician leadership is leadership through medical expertise combined with good manners, collegiality and traits from different kinds of leadership theories.

**Originality/value:**

This study discovers elements of good physician leadership in a Finnish health-care context in which no similar prior empirical research has been carried out.

## Introduction

### Definition of physician leadership

Although leadership is generally defined as “a social process of influence towards the attainment of a common goal, and its task is to achieve direction, alignment and commitment” (Swanwick, 2017, p. 35), similar agreed-upon definitions for physician leadership do not exist in literature. Instead, the terms “physician leadership”, “medical leadership” and “clinical leadership” are often used interchangeably, as if these were synonymous, which creates unwanted confusion regarding what is being referred to. According to Spurgeon *et al.* (2015, pp. 175–176), some authors use the term medical leadership to refer to doctors’ influence processes upon other doctors and clinical leadership as an influence process upon any health-care professional with clinical training. Particularly in American literature, the term “physician leadership” tends to be preferred. Based on an international systematic literature review, physician leaders’ work comprises of both general management/leadership activities and medicine, and medical leadership is defined as what physicians, either with formal managerial roles or acting as informal leaders, do in their daily leadership work to influence others towards goal attainment. Furthermore, the term “medical leadership” is used interchangeably with the term “medical management”, especially referring to physicians’ administrative roles and tasks (Berghout *et al.*, 2017, pp. 1–2).

**Figure 1.**
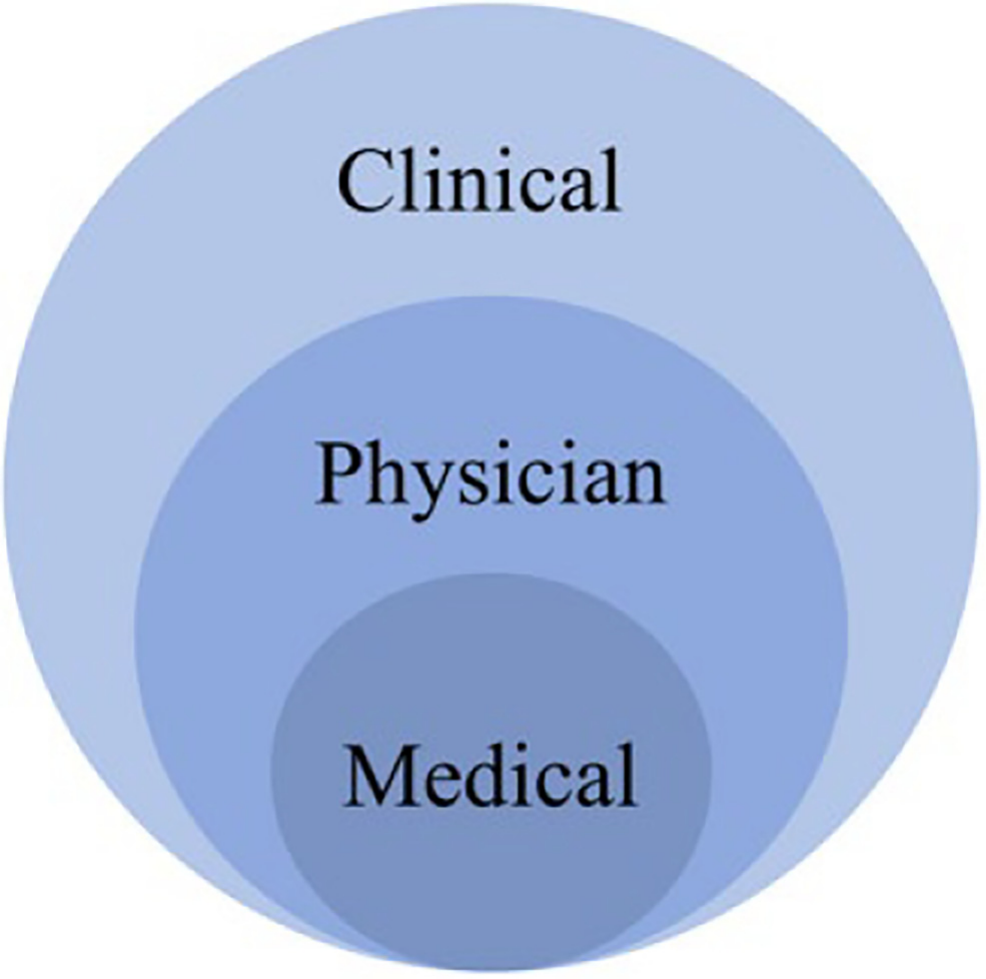
How medical, physician and clinical leadership concepts are embedded

To avoid unwanted confusion, a distinction between the aforementioned terms is made in this study. “Medical leadership” is used to denote activities aimed at managing specific medical knowledge used in patient work, the term “physician leadership” is used to denote leadership activity by a physician leader towards physicians being led, whereas the term “clinical leadership” is seen as a general term referring to leadership activities concerning any group of health-care professionals, rather than specific groups such as physicians. The embedded nature of the concepts is portrayed in Figure 1.

### Elements of good physician leadership

The word good, according to the Oxford Dictionary of English (2021), refers to virtues and is defined as being desired or approved, having the required qualities, possessing or displaying moral virtue, giving pleasure, being enjoyable or satisfying, having a high standard or being thorough or valid.

In contrast, Aristotle (Peters, 1906, p. 1) offers a philosophical take on the issue: “good is that at which everything aims”. Thus, although the concept of “good” may be defined in various ways, any definition would be of little value without context and a reference point. Therefore “good” remains an elusive concept that is abstract in its essence.

Leadership study, as argued by Ciulla (2018, pp. 444–446), is seeking to understand the nature of good leadership wherein ethics and effectiveness overlap. As Ciulla (2018) comments in Aristotelian spirit, a good leader does the right thing in the right way and for the right reasons. Ciulla (2018, pp. 441, 444) further points out that when asking “What is good leadership?”, what is sought after is a demarcation of how leadership ought to be. Therefore, the point of studying leadership is to answer this question where “good” has two senses: morally good and technically good.

In the health-care context, the National Health Service, UK, has delineated the Healthcare Leadership Model consisting of nine core dimensions to help formal and informal leaders in health and care become better leaders. The dimensions are leading with care, evaluating information, connecting our service, sharing the vision, engaging the team, inspiring shared purpose, influencing for results, developing capability and holding to an account (Healthcare Leadership Model, 2013), which are applicable for describing the elements of good physician leadership.

Moreover, recognising there is no one best approach to physician leadership, Oostra (2016) suggests that it should include the following three sets of attributes:

1. belong, believe and build;
2. cascade, connect and champion; and
3. distributed, dyad and development.

The attributes suggest physician leaders should be involved in decision-making, build trust between physician leaders and administrative leaders based on transparent communication, take their physician colleagues through an engagement process with the leadership structure, promoting feelings by all physicians of belonging, which give trust in the system and its functioning capacity. In this kind of environment, physicians can confidently connect with their peers and cascade information, while championing mutually beneficial individual, organisational and community needs and goals. Oostra (2016) suggests a situationally distributed dyad framework in which roles and responsibilities are shared when developing physician leaders to achieve success in the healthcare environment.

The research task of this article is to discover elements of good physician leadership as perceived by physicians with and without formal leadership duties working in the risk-filled occupational context of medicine (Collin *et al.*, 2011) and to find out how the findings connect to the leadership theory.

## Theoretical framework

There are several ways of classifying theoretical approaches to leadership and its study based, for instance, on traits, behaviour, power influence, situational or integrative approach or by types of variables relevant for understanding the effectiveness of leadership, such as the characteristics of leaders, followers or the situation (Bass and Stogdill, 1990, pp. 11–19). To facilitate analysis of the empirical data, the selected leadership theories compiled from a wider set of available leadership theories form the theoretical framework for this study. Even though each leadership theory represents a conception of what good leadership is (Ciulla, 2018, pp. 444–446), their selection for the theoretical framework was driven by relevance and fit in the physician leadership context to allow perceptive analysis.

### Distributed, shared/collective and collaborative theory

This set of theories favour sharing power between leaders and followers and removing the clear-cut boundary separating the identities present in traditional approaches to leadership (Gordon, 2011, pp. 194–195). According to Yukl (2013, pp. 290–291), distributed leadership involves multiple leaders with distinct but interrelated responsibilities, with the leaders’ responsibility to “encourage and enable others to share responsibility for leadership functions”. In shared/collective leadership, the leadership power is perceived as being “wherever the expertise, capability and motivation” happen to be located. Collaborative leadership, on the other hand, presumes that leadership work needs the effort of several individuals who may come from different professions and organisations. Leadership teams are more valued than solo leaders (Swanwick, 2017, p. 45).

### Transformational theory

In transformational leadership, subordinates are empowered by leaders who increase the sense of efficacy and purpose as well as inspire subordinates to work together towards goals (Burns, 2003, p. 26). Subordinates are thought to feel “trust, admiration, loyalty and respect” towards their leader. Transformational leadership is said to appeal to the moral values of the followers. The leader’s goal is then to try to raise their subordinates’ awareness of ethical issues and improve the organisation by putting the subordinates’ energy and resources into motion (Burns, 2003, p. 29; Yukl, 2013, pp. 312–313). Leaders’ behaviour is confident and optimistic, and they state a vision for the organisation and explain how it can be obtained. Dramatic and symbolic actions by leaders are ways to highlight the main values. The leader is perceived capable of influencing subordinates’ commitment to work by setting an example of desired behaviour as part of their interactions with them (Yukl, 2013, pp. 323–325). Transformational leadership is argued to improve subordinates’ experiences and outcomes (Swanwick, 2017, p. 47).

### Ethical and servant theory

It is suggested that ethical leadership manifests in the forms of external guidance and internal commitment (Bowman and Swanwick, 2017, p. 203). Leaders appear truthful, caring and principled when making objective decisions. They set ethical standards and work and behave according to them (Brown and Treviño, 2006). Ethical leaders do not play favourites or support mistrust to gain more power or achieve personal objectives, as they need to encourage ethical behaviour and discourage unethical practices. Ethical leaders are considered to be capable of building mutual trust and respect among subordinates, solving conflicts between different parties and influencing subordinates into realising the need for adaptive problem-solving that will enhance their long‐term welfare (Yukl, 2013, p. 345). Similar to ethical leaders, servant leaders protect and develop all their followers, treating them with equal respect and appreciation. Regardless of the financial interests of the organisation, servant leaders are to stand for what is right and good (Yukl, 2013, pp. 337, 345). Furthermore, the leader is to educate their subordinates through mentoring, coaching and training and remain consistent with the supported values in their behaviour while encouraging critical thinking to find the best alternatives. These theories suggest that leaders need to generate a vision based on subordinate input concerning their ideas, values and needs and take personal risks and actions to fulfil the vision (Yukl, 2013, p. 342).

### Contingency theory and situational theory

Contingency theory centres on understanding how situational aspects modify a leader’s influence on subordinates or workgroups (Yukl, 2013, p. 169) to match the right kind of leader with a situation they can efficiently handle (Gordon, 2011, p. 194). The effectiveness of leaders and organisations does not only depend on the leader’s personality and the degree to which they are task-motivated, but also on the situational control, which clarifies to what extent the situation provides leaders with power, control and influence over the outcome. Task-motivated leaders are more successful in situations of very high or relatively low control, whereas relationship-motivated leaders are more successful in situations of moderate control (Fiedler, 1981). The situational approach to leadership highlights the importance of contextual elements that affect leadership processes (Yukl, 2013, p. 29).

## Data and methods

The subjects of this empirical study were physicians of the Central Finland Health Care District wherein approximately 800 physicians are working (Finnish Medical Association, 2016). The informants were invited and volunteered to participate in the study with an internal email. They form two groups:

1. those with leadership responsibilities hereinafter referred to as physician leaders (15 chief physicians, eight heads of departments); and
2. those without such responsibilities referred to as physicians (13 specialists, 14 specialising physicians/residents).

Although, for the most part, the two groups were treated as representatives of the physician profession, a distinction of the level of the physician category was made if the professional hierarchy level appeared specifically relevant to the findings. Most of the physician leaders were male (14/23), whereas most of the physicians were female (16/27). Chief physicians had on average 11 years of leadership experience, whereas heads of departments had roughly five. As expected, the physician leaders were typically somewhat older (41–62 years) than physicians (26–54 years). All the participants were eligible for the study as the research task was to discover elements of good physician leadership as perceived by members of the doctor’s profession. The study was granted ethics approval by the Central Finland Care District officials. Due consideration was given to matters related to data protection following the ethical principles applicable to research subjects (Finnish Advisory Board on Research Integrity, 2012).

The interviews were conducted in private, typically in the informant’s office during April–June 2017 and July–August 2018. The interviewees were duly informed about the purpose of the study and their right to withdraw their participation and deny the use of their data at any time during the study. The interviews were digitally recorded, transcribed and coded with numbering to make the data more accessible for analysis. The duration of the individual interviews ranged from 11 to 85 min, approximately 25 h in total. The transcribed interviews yielded a total of 619 A4-sized pages (Calibri, 12 point, single spacing).

A qualitative, constructivist-interpretative approach (Gibbs, 2007) was chosen as the research strategy due to the research task involving furthering our understanding of the elements of good physician leadership as perceived by the informants. Semi-structured interviews were chosen as the data collection method. Each informant was asked to describe freely: “What does good physician leadership mean to you?” If the informant understood the question and was able to answer the question, and likewise, the interviewer understood the answer; no further questions were asked. If the informant felt he/she did not understand the question, the interviewer asked the same question in other words. If the interviewer did not understand the answer, the interviewer asked: “Could you explain your answer in other words?”

Systematic, structured content analysis by thematisation was chosen as the method of analysis. Content analysis is based on a systematic examination of the whole set of empirical data with the unit of analysis being for example the individual participants or the whole group. Its focus is on themes and patterns by picking up common or exceptional statements or viewpoints and continuing with identifying and comparing information, groups or subgroups, themes and patterns and meaning. In the end, connections between points of view, themes and patterns of the discussion are theorised. Thematisation is a commonly used technique for organising empirical data in studies based on content analysis, such as this. A theme can be defined as a concept, trend, idea or distinction that emerges from the empirical data (Eriksson and Kovalainen, 2008, pp. 187–189, 219).

The analysis was conducted to identify patterns and themes across the data using a step-by-step procedure. In the first step, the author familiarised herself with the data by reading and immersing herself in the data to the point where the knowledge of the content was firm and deep. In the second step, the author identified the most basic elemental codes, words or phrases that represent the most basic elements of the raw data, based on which the data was organised into groups. In the third step, the themes were searched by grouping the codes into potential themes representing patterns emerging from the data. In the fourth step, the potential themes were reviewed and refined to the point where each theme was internally coherent and there were identifiable distinctions between them. After that, the themes were organised into a thematic table. In the fifth step, the themes were named and distinct definitions for each theme were created. Each theme was analysed separately and within the context of the overall thematic structure. In the last step, the author adjusted the thematic structure as necessary to provide a clear and coherent representation of the data and prepared a report outlining the conceptual ordering with carefully selected excerpts to add validity to the findings and illustrate the interpretations made. Expressions that could have endangered the informant’s anonymity have been removed or replaced in the interview excerpts. These changes did not affect the results because the original transcriptions were used for analysis. Excerpts from chief physicians are marked with (C), those from heads of departments with (H), those from specialists with (S) and those from specialising physicians/residents with (R). As a result, five categories representing elements of good physician leadership (relationship skills, collegiality and physician’s autonomy, medical leadership shown by experience of physician’s work, appropriate leadership approach and working conditions) emerged from the data. As the analysis was data-driven, the whole step-by-step procedure was repeated twice to avoid bias. The author returned to the data several times during the analysis process to confirm that the results truly represent the informants’ voices, and the results were further evaluated against leadership theories particularly connecting physician leadership principles as outlined in the theoretical framework to assess how the elements of good physician leadership relate to them.

## Results

Based on the data, the elements of perceived good physician leadership are based on similar fundamental elements of good leadership as with any other professional group. However, the elements involve certain special aspects related to physician work. The elements and their profession-specific aspects are presented in the following.

### Relationship skills

According to the informants, good manners and mastering basic relationship skills, such as an ability to relate to others in communication and willingness to listen, are important elements of good physician leadership. The skill of empathy and mindset needed to adopt subordinates’ positions were considered important.

A leader may not lose his temper, curse, stab in the back, be unfair or build inner circles in which some are in a better position than others […] Impartiality, tactful conduct and matter-of-factness are an integral part of good leadership. (C11)

Communication cannot be based on the assumption that leaders know what their subordinates think and vice versa. Mutual and reciprocal continuous communication between leaders and followers was seen as a precondition for all actions, and the low threshold for presenting one’s thoughts and ideas to one’s superior was perceived to be important. Engaging physicians in decision-making concerning them was considered a reasonable and easier way to succeed in leadership.

I do not necessarily make my own decisions without first listening to and discussing with other physicians […] especially when larger issues are concerned, they need to be discussed first and then come up with the decision. (C15)

Leaders’ communication may not only be a transmission of information, but instead, informants felt that showing interest towards subordinates’ work and how they are coping was a good leadership practice. Informants felt it more natural to refer to physicians being lead as “physicians” rather than “subordinates”, and in mutual discussions, irrespective of the hierarchy level, the use of first names was preferred over the use of job title alone or in combination with a surname.

The informants hoped that performance and development appraisals would take place on an annual basis, and specialising physicians wanted to receive “signposts” to their careers in them. Leaders were regularly expected to provide constructive feedback, both positive and negative. Leaders themselves also desire feedback regarding their work from their superiors but also appreciate it from subordinates.

My supervisor has been interested in the direction I wish to develop in my work or career as a physician […] and he pitched ideas about the potential direction my career as a physician could take. (S19)

The way the feedback is given must be carefully considered. (H23)

Feedback would be the most important thing, that the leader would also receive feedback from their superior. (C44)

Feedback was best given face-to-face, and positive feedback was not given often enough according to the informants. The trickier the leadership issue was, the more physicians expected face-to-face encounters. The ability to work undisturbed was perceived as a form of positive feedback and a sign of trust in the physician being competent and capable of succeeding in their work.

Good physician leadership involves an element of trust […] if everything works, then things can be left as they are, trusting that these guys will finish the job properly […] I think allowing people to work undisturbed is, as such, already a form of positive feedback. (R8)

Negative issues must be dealt with quickly and as forthright as possible […] not through e-mail discussions, but preferably through telephone calls or face-to-face conversations […] straight away, fairly, and impartially. (C11)

The word “feedback” was felt to have a somewhat negative connotation and was associated with a failure in one’s work. However, being truthfully informed face-to-face about one’s shortcomings in work was considered a feature of good leadership.

The informants expected situational predictability in leaders’ behaviour: calmness and consistency. The informants felt it important that their leaders were responsible for their leadership and patient work, appreciative of the work of the physicians they lead and equitable and just towards them. Playing favourites was considered unacceptable. The informants expected leaders to possess situational awareness and emotional intelligence to present things in a well-reasoned, non-offensive way.

The capacity for empathy and compassion are also an essential part of good physician leadership, and if they are missing, management is unlikely to work. (H10)

Decision-making ability and courage to address shortcomings, such as inequalities in subordinates’ workloads, were considered necessary qualities in leaders. Information about who made the decisions clarified the division of responsibilities and reinforced a sense of safe working.

Courage to make even unpleasant decisions and take up issues that are not easy to address. (S22)

Physicians as subordinates were regarded as a distinctive, highly educated and self-aware group of experts with strong personalities and opinions. For this reason, leaders must get along with different kinds of people and understand their strengths and weaknesses.

Good physician leadership requires a certain kind of authority, but then again, humility to listen and negotiate is also necessary. It also requires diplomacy skills because this is a large organisation, in which relationships and community-building skills are needed. (H40)

Leaders, as senior physician colleagues, were considered partly responsible for shaping the behavioural culture of the workplace due to the expectation of demonstrating what appropriate behaviour in the workplace was.

A leader’s task is to create the preconditions for successful work performance and to be able to maintain a positive atmosphere of doing things together. (C35)

Occasionally, informants felt it was forgotten that physicians are also people with feelings and emotions, which emphasises the need for interpersonal skills in physician leadership.

### Collegiality and physicians’ autonomy

The informants routinely included collegiality as an element of good physician leadership. Collegiality was perceived to manifest as general appreciation towards colleagues and respect for another’s work, but also as a certain kind of balance that manifested in the form of equality between colleagues.

When you lead, you have to be collegial in the same way as physicians are collegial towards one other, and this also applies to a physician leader. (S49)

In challenging situations, good leadership appeared as clear and collegial guidance in which leaders backed up their people rather than primarily serving their interests.

Even though physicians are colleagues to one another regardless of the hierarchical level, a certain distance between physician leaders and subordinates was felt necessary, and therefore, closer friendships were not seen as desirable or necessary. However, too much distance was perceived to result in talking behind backs and the formation of small cliques, complicating leadership.

[A good leader] is friendly and matter-of-factly but does not attempt to be buddies with you. Remembers his position. (H41)

For successful leadership, it was considered practical to be able to liaise with the physicians being led during working days in neutral situations (e.g. in the hospital cafeteria/canteen/corridor) in addition to during patient work. In that way, leaders provided subordinates an opportunity to speak their minds. Some leaders considered being readily available, responding to work calls even when off-duty, for example, as a sign of responsibility and dependability which was required for good leadership. However, controlling work too closely was perceived as lacking confidence in the subordinate’s professional ability and autonomy, which, in turn, was perceived as undermining the subordinate’s respect for the leader.

As experts, we [physicians] demand or need our freedom to act […] distrustful monitoring will not do. (S25)

I trust the physicians I lead, and I dare to let the physicians in my clinic do the job they’ve been hired to do. (H1)

I’m one of those 24/7 leaders, always available […] I am extremely committed to the working community and my team. That’s what a good leader is like. (C11)

According to the informants, good physician leadership meant giving enough freedom to subordinates: not intervening with minor details and allowing them to work undisturbed by trusting their expertise in their field and capability at their job.

### Medical strenuous process the physicians have already gone through leadership demonstrated by experience

Good physician leadership was not perceived as something that could be learned solely through leadership studies, nor was medical training alone seen as a self-evident guarantee of success. Partly, it was seen as something physicians grow into, similar to physicianship. The informants perceived a physician’s profession unique under the Hippocratic Oath not only as a profession but also as a lifestyle. Therefore, when leading physicians, it was considered a necessity that leaders themselves had gone through the same education and working experiences and had grown to understand the importance of doctor’s ethics in their work.

A good physician leader understands the whole process that is part of the work as a physician[…] what medical training really means and what kind of strenuous process the physicians have already gone through before they enter working life. The leader understands the healthcare system […] and the pressure under which physicians do their work and how much on-call duty the work involves. (C36)

In physician leadership, leadership was earned by a leader’s own conduct and working performance. An academic leadership/management title alone was not sufficient to convince physicians about leadership capabilities.

Leadership must be earned, trust comes through actions […] It is not enough to just declare that my values as a physician leader are fairness and impartiality and that I am capable of making decisions. Leadership competence must be proven with actions as part of everyday work. (C35)

The informants perceived medical credibility as an essential element for good physician leadership, and it was sought after by doing patient work to establish one’s competence. Some leaders perceived self-sacrificing behaviour as a sign of good leadership that raised a sense of duty among subordinates. For example, if there was a shortage of physicians for an on-call shift at an emergency clinic, the physician leader did the shift himself.

Good physician leadership is leading by one’s own work – by showing the subordinates that this is how the physician’s work should be done. (C11)

In hospital settings, specialties had their distinct features affecting physicians and their work which could not be ignored in physician leadership.

The basic requirement is that specialist medical competence must be mastered. Otherwise, the subordinates will lead the leader with their substance competence. (H10)

If the leader doesn’t know the substance, he will not be able to make decisions without substance experts' help, which in turn may lead to a situation where the leader doesn’t quite know who to believe. A leader who knows the field is much better able to filter and evaluate information. (H23)

Based on the findings, the leaders of specialists had to master specialist work in the field, and a sufficiently strong medical background was deemed necessary, as leaders were otherwise felt to be disadvantaged in their leadership work and lacking credibility in the eyes of their subordinates.

### Appropriate leadership approach

The ability to be a leader for each individual employee and take the situation at hand into account was considered important elements of good physician leadership. The informants presumed physician leaders to possess – through their previous medical and working career – the knowledge, skills and understanding of the support needed by physicians at different career stages.

There is not only one kind of good physician leadership; instead, there are many kinds of good physician leadership. (C11)

The current career/specialisation stage and the underlying life experience of the physicians being led were important aspects to consider because physicians at different stages of their career need different kind of leadership. In the early stages of physicians’ careers, difficult situations were more frequently experienced in patient encounters, which was why young physicians needed mental support from their supervisor in addition to the physical presence of a leader.

A physician just starting his specialisation training is in a completely different situation […]compared to a situation where the physician has already worked as a specialist for years. (H6)

Some differences of opinion regarding the leader’s relationship with the physicians being led arose among the informants. Some thought that the key factor in successful physician leadership was physicians being led from the front line. However, autocratic leadership was perceived as old-fashioned and a sure way to fail in physician leadership. Dictating orders to highly educated people was not deemed viable, but instead, well-founded reasons were expected to exist when giving orders. Some of the informants perceived good physician leadership as a service profession in which leadership is bottom-up leadership.

A good physician leader leads his troops if not exactly from the front line, then at least very close to it. (H1)

Informants considered physicians as leaders of their work and of multi-professional teams they worked with, highlighting the informal leadership in the physician profession. Therefore, formal leaders were expected to be able to dial back responsibility and dare to give the physicians – as informal leaders – the opportunity to direct themselves if the working community functions well.

If you have responsibility without power, nothing will work. And if you have power without responsibility, it will not work either. (H6)

Integrating elements of the business world as part of physician leadership was not considered possible because of ethical issues related to the field of medicine guiding the work of all physicians, irrespective of the hierarchical level.

We can’t be quite that tough and just focus on the figures. In our field, people and things are managed, and on the other hand, we are also really often at this interface of humanity, as it were. (R20)

For the success of physician leadership, it was considered important that power, responsibility and ethics went hand in hand within the same leader.

### Working conditions

An important aspect of good physician leadership for physicians was their work running smoothly; everyday work should not be day-to-day survival. Knowledge of health-care systems, foresight skills and a vision of the direction being pursued were necessary virtues of leaders for enabling hassle-free work. The construction of leaders’ visions needed to be encouraging and forward-looking in a determined way.

That [leadership] is not just living in the moment. The leader must have an idea of where the healthcare system is going and what is being sought. (C27)

Smoothly running work ensured that physicians were satisfied with their work and would not seek work elsewhere. This was achieved with a well-functioning framework for working: appropriate employment contracts, compliance with working conditions and hours, properly assigned responsibilities, opportunity to work undisturbed and ensuring that all new physicians, (regardless of the hierarchical level) were also able to start their work smoothly with a proper induction.

The physician leader takes the unit’s side and sees to it that there are sufficient resources available and that the employees can do their work now, and in the future. (S38)

There should be induction, and you should be told who your supervisor is and who you can turn to if you encounter problems. (R12)

Physicians voted with their feet if not satisfied with their working conditions and/or their leader’s leadership skills.

To make employees want to stay, a physician leader must have the ability to see the situation and the big picture. If a physician is planning to leave, this needs to be addressed rather quickly, asking what we should do to keep you here. (H23)

Based on the findings, leaders would need to sense situations that could result in departure and be humble enough to bring potential leaving plans up for discussion and to persuade physicians to stay in their current position.

## Discussion

As noted above, leadership is conceptualised as “a social process of influence towards the attainment of a common goal, and its task is to achieve direction, alignment and commitment” (Swanwick, 2017, p. 35). Based on the results of this study, these features are also found in physician leadership but are not enough to succeed by themselves. According to the findings of this study, the elements of good physician leadership perceived by physicians include relationship skills, collegiality, physician’s autonomy, medical leadership demonstrated by the experience of physician’s work, appropriate leadership approach and working conditions, which are parallel to previous studies’ findings (Healthcare Leadership Model, 2013; Oostra, 2016; Ciulla, 2018, pp. 441, 444).

In the following section, the results of this study are reflected against the distributed, shared/collective and collaborative theory, transformational theory, ethical and servant theory and contingency theory and situational theory.

### Leading peers – leading leaders

According to the findings, physician leaders were not only leaders but also physician colleagues of their subordinates because, as shown in this study, most participated in patient work, which blurs the leader–follower boundary. Success in medical leadership came from credibility in medical expertise, which made medical leadership an inseparable part of physician leadership. For example, if some advice were needed in difficult medical or patient situations, specialists with extensive medical career and life experience still expected back-up from their leaders and/or a second opinion from their peers. This exemplifies the informal and shared/collective nature of leadership in the physician profession. Futhermore, this extends to physician leaders as they participated in patient work alongside their leadership work, signifying that physicians could generate a mutual medical leadership regardless of the hierarchical level through medicine. As the informal leadership role associated with a physician’s work turns all physicians into leaders on their hierarchical level, the physician leadership is a multidimensional leadership setting in which formal leaders led informal leaders. This further blurs the boundaries between followers and leaders. These professional traits are probably part of the reason why physicians found it hard to accept anyone other than physicians as their leaders.

In sum, these profession-specific features – instilling leadership responsibility to a wider body of organisational members than only those at the top – support distributed, shared/collective and collaborative leadership as an important approach for constructing good physician leadership as this set of theories favours sharing power between leaders and followers and removing the clear-cut boundary separating the identities present in traditional approaches to leadership (Gordon, 2011, pp. 194–195).

### Physicians’ autonomy and working conditions

Based on the findings, physicians expected good physician leadership to establish good role model behaviour and recognise the autonomy of a physician’s work. Activities that streamline work were deemed to be a part of good physician leadership. The beneficial attributes of a leader include trust, fairness, empathy, social skills, two-way communication skills, regular feedback, collegial respect and emotional intelligence. Good manners are considered a necessity. These findings are similar to what previous studies have found (Healthcare Leadership Model, 2013; Oostra, 2016). According to the findings, when referring to the physicians being led, the terms “subordinate” and “employee” were not recommended by physicians at any level. The informants preferred subordinates/employees being called “physicians” when referring to them as a group. In other situations, first names were preferred, but not titles nor titles with surnames.

Physician leaders were expected to be capable of making decisions and to have visionary talents. Natural-born leaders were not expected, and a job title was not perceived enough to earn credibility as a leader to subordinates and peers. The status of a respected leader was earned by showing not only capability for leadership but also an understanding of medicine and a physician’s work and life. This was assumed to be difficult without prior knowledge of working as a physician because it takes medical education and years of work experience to properly internalise medical ethics and the responsibilities of physicians. A meaningful vision for the organisation and hassle-free working conditions were seen to contribute positively to the desired level of job satisfaction.

In short, these features support the transformational leadership theory as a good ground to build a good physician leadership as leaders’ behaviour was required to be confident and optimistic, and they needed to have a vision for the organisation in addition to being capable of explaining how to obtain it. The transformational leader was perceived to be capable of influencing subordinates’ commitment to work by setting an example of desired behaviour as part of their interactions with them (Yukl, 2013, pp. 323–325). This was perceived to occur naturally in patient work alongside the leadership work, as physician leaders did and were expected to participate in clinical patient work according to the findings of this study. This also helped physician leaders to understand and to respect the physician’s autonomy, which caused them to feel trust, admiration, loyalty and respect towards their leader, which are intrinsic parts of the transitional leadership approach (Burns, 2003, p. 29; Yukl, 2013, pp. 312–313).

### Common ethical goal

The physician’s profession and the context of leading fellow physicians set a specific tone and expectation for the leadership approach. Physicians had a strong internal commitment to accomplish their work with ethical behaviour. This study corroborated the view that physicians wanted to be led by a leader with the same internalised ethical and medical values (Styhre *et al.*, 2016) combined with mutual trust and collegial and professional respect, which reflected ethical leadership. Collegial and communal obligations calling to cherish and help one’s peers and strive to do no harm, only what is right and good, served as a basis of good physician leadership. Thus, the Hippocratic Oath of the physician profession brought about the need for ethical leadership and servant leadership in particular.

To summarise, these features support the importance of ethical leadership as part of good physician leadership. Physician leaders needed to show ethical standards via their work and behave according to what they believe in (Brown and Treviño, 2006). Moreover, following Aristotle (Peters, 1906, p. 2), the physician leader was to promote equally good treatment for followers and could not play favourites if acting in Aristotelian good leadership spirit. This also reflected the servant leadership approach as physician leaders were expected to educate subordinates as peers through mentoring, coaching and training and remain consistent with the supported values in their behaviour, while encouraging critical thinking to find the best alternatives (Yukl, 2013, p. 342).

### Different needs between hierarchical levels

Because of the different hierarchical levels in the physician profession, the contingency theory is readily applicable to good physician leadership. The situational approach highlights the importance of the contextual elements that affect the leadership processes. The findings of the study showed that the needs of hierarchical levels were different to a certain extent, even though there were many similarities as well. In general, fairness, collegial behaviour and feedback on work were taken to represent good physician leadership. However, there were also needs specific to each level. Based on the leader’s prior work history as a physician and career as a leader, subordinates expected leaders to understand suitable ways to lead in different situations. The expected social skills, combined with emotional intelligence and situational awareness, were believed to help leaders find a suitable approach to leadership, with appropriate work and social distance in each case.

Specialising physicians/residents wanted and needed more medical leadership in the form of teaching and consultation help. They also desired career advice as well as role model support during their growth into doctorhood. Unlike specialising physicians/residents, specialists already had a long medical career and lots of life experience underline the significance of hassle-free working conditions and expected an understanding of the autonomy of a specialist’s work as an important element of good physician leadership. Because the medical knowledge of specialists was at a high level, they usually did not need medical advice in the same way as specialising physicians/residents. At the level of formal physician leaders, good physician leadership was perceived as a situation in which subordinates succeeded in their work and the leader could provide enough physicians for the clinic and safeguard the necessary work equipment to ensure smoothly running working conditions. Physician leaders in formal leadership positions desired feedback on their leadership work from their superiors, but also appreciated it from their subordinates. Preferably, feedback was received in person.

In essence, these features support the contingency and situational approach as building blocks of good physician leadership as the contingency theory centres on understanding how situational aspects modify a leader’s influence on subordinates or workgroups (Yukl, 2013, p. 169) to match the right kind of leader with a situation they can efficiently handle (Gordon, 2011, p. 194). Furthermore, the situational approach highlights the importance of contextual elements that affect leadership processes (Yukl, 2013, p. 29).

## Conclusion

Based on the findings, good physician leadership is multi-theoretical. As subordinates are physician colleagues following the Hippocratic Oath and having informal leadership roles on their respective hierarchical levels, the physician leadership is a multidimensional leadership setting wherein formal leaders lead informal leaders, which blurs the traditional leader–follower boundary, which has an impact on how good physician leadership is perceived. As medical expertise connects leaders and followers, success in medical leadership comes from credibility in medical expertise, making medical leadership an inseparable part of physician leadership, but not synonymous with it. Good physician leadership is thus leadership through medical expertise, combined with good manners, collegiality and traits appearing in various leadership theories. Most often, different approaches are needed concurrently. The most suitable approach to combining them depends on the situation and the hierarchical level on which physicians are led and physician leaders are working. Ethical, transformational, contingency and situational aspects of leadership are present on every hierarchical level as part of good physician leadership. Physician leaders also need a distributed, shared/collective, collaborative and servant leadership approach to accomplish their leadership work. Physician leaders are expected to possess the professional skills of physicians, understand how the work affects physicians’ lives and be competent in applying suitable leadership approaches per different situations and people. Trust, fairness, empathy, social skills, two-way communication skills, regular feedback, collegial respect and emotional intelligence are expected.

## Limitations and further research

This study was limited to a single hospital district, the Central Finland Health Care District. However, it represents the largest non-university hospital district in Finland (Association of Finnish Municipalities, 2020). In further research, it would be worthwhile to investigate physician leadership at the university hospital level and compare perceptions between the different levels.
